# Clinical Characteristics and Five-Year Outcomes of Patients Aged 75 Years or Older Receiving Maintenance Hemodialysis at a Japanese Regional Core Hospital

**DOI:** 10.7759/cureus.111679

**Published:** 2026-06-28

**Authors:** Koji Onozawa, Takuya Kishi, Ayako Takamori, Takashi Muto, Miki Sugiyama, Arisa Kamei, Akira Kitajima, Tomohiro Imamura, Kazuma Fujimoto, Motoaki Miyazono

**Affiliations:** 1 Department of Nephrology, Kouhou-kai Takagi Hospital, International University of Health and Welfare Graduate School of Medicine, Okawa, JPN; 2 Department of Cardiology, International University of Health and Welfare Graduate School of Medicine, Okawa, JPN; 3 Department of Biostatistics, Clinical Research Center in Hiroshima, Hiroshima University, Hiroshima, JPN; 4 Department of Nephrology, Kouhou-kai Takagi Hospital, Okawa, JPN; 5 Department of Neurology, International University of Health and Welfare Graduate School of Medicine, Okawa, JPN; 6 Division of Gastroenterology, International University of Health and Welfare Graduate School of Medicine, Okawa, JPN; 7 Department of Nephrology, Saga University, Saga, JPN

**Keywords:** bone fracture, cardiovascular disease, infection, japanese geriatrics, mortality

## Abstract

Introduction

The number of older patients receiving maintenance hemodialysis is increasing in Japan. Patients aged 75 years or older often have multimorbidity, frailty, nutritional vulnerability, and a higher risk of adverse clinical outcomes than younger patients. However, real-world longitudinal data from regional hospitals regarding mortality and clinically important complications in this population remain limited. This study aimed to compare five-year mortality, baseline clinical characteristics, laboratory findings, GI bleeding, and fractures between maintenance hemodialysis patients aged 75 years or older and those younger than 75 years.

Methods

This retrospective, single-center, longitudinal observational study included all eligible patients receiving maintenance hemodialysis at Takagi Hospital, a regional core hospital in Fukuoka, Japan, on December 1, 2017. Patients were categorized by baseline age into an older group aged 75 years or older and a younger group younger than 75 years. Baseline demographic, clinical, and laboratory data were extracted from electronic medical records. Follow-up continued until death or December 2022. The primary outcome was five-year all-cause mortality. Secondary outcomes included cause-specific mortality, endoscopy-confirmed GI bleeding, imaging-confirmed fractures, and baseline laboratory parameters. Between-group comparisons were performed using Student’s t-test for continuous variables and the chi-square test for categorical variables.

Results

A total of 151 patients were analyzed, including 57 patients aged 75 years or older and 94 patients younger than 75 years. Five-year all-cause mortality was significantly higher in the older group than in the younger group (34/57 (59.6%) vs. 21/94 (22.3%), P < 0.001). Deaths due to renal failure and infection were also more frequent in the older group (21.1% vs. 6.4%, P = 0.007; and 19.3% vs. 5.3%, P = 0.007, respectively), whereas cerebrovascular/cardiovascular mortality did not differ significantly between groups (7.0% vs. 8.5%, P = 0.742). Upper-body fractures occurred more frequently in the older group than in the younger group (21.1% vs. 6.4%, P = 0.007), while lower-body fractures and gastrointestinal bleeding showed no statistically significant differences. The older group had significantly lower serum albumin and phosphorus levels than the younger group.

Conclusion

Among maintenance hemodialysis patients treated at a single regional Japanese hospital, a baseline age of 75 years or older was associated with higher five-year all-cause mortality, more frequent upper-body fractures, and lower serum albumin and phosphorus levels. These findings suggest that older hemodialysis patients may require individualized, multidisciplinary care focused on nutritional status, infection prevention, fracture and fall risk, and supportive care. Because this was a retrospective single-center study, further multicenter prospective studies are needed to validate these findings and clarify targeted interventions for this vulnerable population.

## Introduction

In Japan, the population receiving maintenance hemodialysis is aging, and patients aged 75 years or older now constitute an increasingly large proportion of the chronic dialysis population [1-4]. This age group differs clinically from younger dialysis patients because advanced age is often accompanied by multimorbidity, frailty, reduced physical reserve, malnutrition or inflammation, and different priorities regarding life-prolonging treatment and quality of life [5-13]. Age-specific data on prognosis and clinically important complications are therefore needed to support individualized dialysis care and shared decision-making [5, 6].

Previous registry and cohort studies in Japan and other countries have consistently shown that older age is associated with higher mortality among patients receiving maintenance hemodialysis [1-3, 14, 15]. However, the applicability of these data to routine practice in regional hospitals remains limited. Large registry studies provide valuable population-level estimates but often have limited detail on clinical events documented during daily care, such as GI bleeding, fractures, and cancer-related outcomes. Conversely, hospital-based studies can provide more granular clinical information, but many have focused on the overall dialysis population, selected outcomes, or short-term prognosis. Thus, longitudinal regional hospital data remain limited, particularly for five-year mortality and clinically important complications among patients aged 75 years or older receiving maintenance hemodialysis.

We used 75 years as the age threshold because this cutoff is commonly used to define late-stage older adults in Japanese geriatric and healthcare settings [4]. This threshold was used only to stratify patients who were already receiving maintenance hemodialysis; it was not intended to define an age at which hemodialysis should be initiated or withheld. In older patients with advanced kidney failure, decisions regarding dialysis initiation or conservative kidney management depend on comorbidities, frailty, functional status, expected prognosis, and patient preferences rather than chronological age alone [5-9]. Patients managed conservatively without hemodialysis were excluded from the present study.

The primary objective of this study was to compare cumulative five-year all-cause mortality between maintenance hemodialysis patients aged 75 years or older and those younger than 75 years at a regional core hospital in Japan. The secondary objectives were to compare baseline clinical characteristics, laboratory findings, incident GI bleeding, incident fractures, and cause-specific mortality between the two age groups. We hypothesized that patients aged 75 years or older would have greater clinical vulnerability, different laboratory profiles, more clinically important complications, and higher cumulative five-year all-cause mortality than younger patients treated in the same dialysis center.

## Materials and methods

Study design and setting

This was a retrospective, single-center, longitudinal observational study conducted at the Kidney and Dialysis Center of Takagi Hospital, a 506-bed regional core hospital in Okawa, Fukuoka, Japan. The hospital provides emergency care and a broad range of specialty services, with an average of 710 outpatients and 387 inpatients per day in fiscal year 2024. The center operates 73 dialysis units, including 61 units in the dialysis center and 12 units in an in-ward dialysis room, and provides maintenance hemodialysis from Monday through Saturday, including night dialysis on Mondays, Wednesdays, and Fridays.

A retrospective prevalent cohort design was selected because the objective was to describe real-world, age-stratified outcomes in the complete maintenance hemodialysis population treated at this center over a fixed observation period using routinely collected clinical data. This design is suitable for describing regional practice but is susceptible to selection bias, information bias, survivor bias, residual confounding, and limited generalizability. Therefore, all eligible patients on the dialysis roster who were seen consecutively were included, and all analyses were interpreted as descriptive and hypothesis-generating rather than causal.

Study population

The study population was defined using the dialysis roster at Takagi Hospital on December 1, 2017, which served as the index date. We screened all patients with end-stage kidney disease who were receiving scheduled maintenance hemodialysis at Takagi Hospital on that date. Maintenance hemodialysis was defined as chronic hemodialysis for end-stage kidney disease performed on a regular schedule; temporary hemodialysis for acute kidney injury and short-term guest dialysis were not considered maintenance hemodialysis.

The inclusion criteria were as follows: (1) receiving maintenance hemodialysis at Takagi Hospital on December 1, 2017; (2) available baseline clinical and laboratory data around the index date; and (3) available follow-up information after the index date. No minimum dialysis vintage was required, and patients were included regardless of dialysis duration or inpatient/outpatient status if they were receiving maintenance hemodialysis on the index date. Patients were excluded if they were receiving temporary hemodialysis for acute kidney injury, peritoneal dialysis without maintenance hemodialysis, dialysis primarily managed at another facility, or had insufficient records to determine baseline age or follow-up status.

Because all eligible patients on the dialysis roster on the index date were included, this study used complete enumeration of prevalent maintenance hemodialysis patients at this center rather than convenience sampling. After applying the eligibility criteria, 151 patients were included in the analysis.

Follow-up and age categorization

Follow-up began on December 1, 2017, and continued until death or the end of the observation period in December 2022. All-cause mortality and cause of death were ascertained from electronic medical records, dialysis records, hospital records, death certificates, discharge summaries, and referral information when applicable. Vital status was available for all patients during the study period.

Age was calculated on the index date. Patients were categorized a priori into an older group aged 75 years or older and a younger group younger than 75 years. Patients remained in their baseline age category throughout the follow-up period, even if they reached 75 years of age during the study period, because age group was treated as a baseline exposure variable. This classification resulted in 57 patients in the older group and 94 patients in the younger group.

Data collection and variable definition

After cohort identification, study variables were retrospectively abstracted from electronic medical records by longitudinal chart review using the definitions described below. Baseline variables were collected from records around December 2017, whereas follow-up events and vital status were identified from records through December 2022, death, or the last documented contact at Takagi Hospital.

Baseline demographic data, dialysis vintage, primary cause of end-stage renal disease, and routine laboratory measurements were extracted from electronic medical records. The primary cause of renal failure was determined from the diagnosis recorded at dialysis initiation and from nephrology notes. Causes were classified as diabetes mellitus, nephrogenic disease, hypertension, or other/undetermined. Diabetes mellitus was assigned when diabetic nephropathy was recorded as the principal cause of end-stage renal failure. Nephrogenic disease was assigned when a non-diabetic renal disease, such as chronic glomerulonephritis, nephrosclerosis, or another primary renal disease, was recorded as the principal cause. Histories of hypertension and diabetes mellitus as separate baseline comorbidities were not uniformly retrievable using standardized retrospective definitions and therefore were not analyzed as independent comorbidity variables.

Routine outpatient monitoring, including blood sampling, was performed approximately monthly. For consistency in this prevalent cohort, laboratory measurements obtained in December 2017 were used as baseline values. These included hemoglobin, total protein, albumin, cholesterol, corrected calcium, intact parathyroid hormone, serum iron, ferritin, and serum phosphorus. Baseline phosphate-binder prescriptions and adherence could not be uniformly abstracted with sufficient accuracy across the entire cohort. Therefore, phosphate-binder exposure was not included as an analytic variable, and the interpretation of serum phosphate levels was limited accordingly.

Gastrointestinal bleeding during follow-up was defined as endoscopy-confirmed upper or lower gastrointestinal bleeding. Endoscopic evaluation at Takagi Hospital was used for confirmation when gastrointestinal bleeding was clinically suspected. Bone fracture was defined as a new fracture documented during follow-up, diagnosed by the orthopedic department, and confirmed by imaging. Plain radiography was the primary confirmatory modality; CT or MRI was used when additional confirmation was required. Clinically suspected fractures without imaging confirmation were not counted.

Fractures were classified as upper-body or lower-body fractures according to the anatomical site recorded in orthopedic records. Upper-body fractures included humeral, radial, and related upper-extremity fractures. Lower-body fractures included femoral neck, intertrochanteric or subtrochanteric femoral fractures, and vertebral compression fractures. Cause-of-death categories were renal failure, infection, cerebrovascular/cardiovascular disease, other causes, and unknown cause. Cause of death was determined from routine clinical documentation; no independent blinded adjudication committee or external adjudication process was used.

Newly diagnosed malignant disease during follow-up was summarized descriptively as an exploratory finding. Malignancy information was obtained from electronic medical records and, when available, specialist documentation. The illness period was defined as the interval from cancer diagnosis to death or the end of follow-up.

Outcomes

The primary outcome was cumulative five-year all-cause mortality. Secondary outcomes were baseline clinical and laboratory characteristics, incident endoscopy-confirmed GI bleeding, incident imaging-confirmed fractures, and cause-specific mortality. Malignant disease during follow-up was evaluated descriptively as an exploratory outcome.

Statistical analysis

Continuous variables are presented as mean ± SD, and categorical variables are presented as n/N (%). Continuous variables were compared between the two baseline age groups using Welch’s t-test. Categorical variables were compared using the chi-square test or Fisher’s exact test when expected cell counts were small. The distribution of the primary cause of renal failure was evaluated as a multi-category variable. All P values were two-sided, and P < 0.05 was considered statistically significant.

Mortality was evaluated as cumulative all-cause and cause-specific mortality during the observation period. Kaplan-Meier curves, Cox proportional hazards models, and competing-risk models were not performed because the study was designed as a descriptive cumulative-outcome analysis and because the modest sample size and limited number of cause-specific deaths would have produced unstable estimates. Similarly, multivariable models were not used to draw conclusions about independent risk factors because important confounders, including frailty, functional status, smoking status, medication exposure, inflammatory activity, dialysis adequacy, treatment preferences, and socioeconomic factors, were not uniformly available. Missing data were handled by available-case analysis, and no imputation was performed. Statistical analyses were performed using JMP Pro 16 (SAS Institute Inc., Cary, NC, USA).

Ethics

This study was conducted in accordance with the Declaration of Helsinki and was approved by the Kouhou-kai Ethics Committee of Takagi Hospital (KR226) and the International University of Health and Welfare Ethics Committee (23-Ifh-48). For the retrospective analysis of existing clinical data, the requirement for written informed consent for study participation was waived by the ethics committees, and information about the study was disclosed with an opt-out option in accordance with institutional procedures. Written informed consent for maintenance hemodialysis treatment was obtained from all patients as part of routine clinical care.

## Results

A total of 151 maintenance hemodialysis patients were included: 57 were aged 75 years or older, and 94 were younger than 75 years at baseline. Baseline clinical characteristics, complications, and cumulative deaths are summarized in Table 1. Sex distribution did not differ significantly between groups. Hemodialysis vintage was also similar between groups. The distribution of the primary cause of renal failure differed between groups, with diabetes mellitus more common in the younger group and hypertension more common in the older group.

**Table 1:** Characteristics of patients aged 75 years or older receiving maintenance hemodialysis were compared with those of younger patients. Continuous variables were compared using Student’s t-test; categorical variables were compared using the chi-square test. Data are presented as mean ± SD or n/N (%). Fractures indicate imaging-confirmed fractures diagnosed by the orthopedic department; deaths indicate cumulative deaths during the five-year observation period. CCD: Cerebrovascular/cardiovascular disease.

Variable	≥75 years old (n = 57)	<75 years old (n = 94)	Test statistic	P value
Male sex	31/57 (54.4%)	59/94 (62.8%)	χ² = 1.03	0.309
Age, years	81.5 ± 9.7	61.0 ± 5.0	t = 17.11	<0.001
BMI, kg/m²	21.9 ± 4.7	20.5 ± 3.3	t = 2.15	0.033
Duration of hemodialysis, months	90.1 ± 97.3	115.2 ± 107.1	t = -1.44	0.151
Primary cause of renal failure				
Diabetes mellitus	13/57 (22.8%)	41/94 (43.6%)	χ² = 5.81	0.0138
Nephrogenic diseases	22/57 (38.6%)	27/94 (28.7%)	χ² = 1.16	0.2158
Hypertension	19/57 (33.3%)	14/94 (14.9%)	χ² = 6.03	0.0139
Upper GI bleeding	5/57 (8.8%)	19/94 (20.2%)	χ² = 3.47	0.062
Lower GI bleeding	3/57 (5.3%)	4/94 (4.3%)	χ² = 0.08	0.775
Upper-body fracture	12/57 (21.1%)	6/94 (6.4%)	χ² = 7.27	0.007
Lower-body fracture	10/57 (17.5%)	13/94 (13.8%)	χ² = 0.38	0.538
Death due to renal failure	12/57 (21.1%)	6/94 (6.4%)	χ² = 7.27	0.007
Death due to infection	11/57 (19.3%)	5/94 (5.3%)	χ² = 7.32	0.007
Death due to CCD	4/57 (7.0%)	8/94 (8.5%)	χ² = 0.11	0.742
Total deaths	34/57 (59.6%)	21/94 (22.3%)	χ² = 19.75	<0.001

Upper-body fractures occurred more frequently in the older group than in the younger group (12/57 (21.1%) vs. 6/94 (6.4%); P = 0.007). Lower-body fractures did not differ significantly between groups (10/57 (17.5%) vs. 13/94 (13.8%); P = 0.538). Upper GI bleeding was numerically more frequent in the younger group (5/57 (8.8%) vs. 19/94 (20.2%); P = 0.062), whereas lower GI bleeding was uncommon in both groups.

Cumulative all-cause mortality during follow-up was higher in the older group than in the younger group (34/57 (59.6%) vs. 21/94 (22.3%); P < 0.001). Death attributed to renal failure (12/57 (21.1%) vs. 6/94 (6.4%); P = 0.007) and death attributed to infection (11/57 (19.3%) vs. 5/94 (5.3%); P = 0.007) were more frequent in the older group. In contrast, cumulative death attributed to cerebrovascular/cardiovascular disease did not differ significantly between groups (4/57 (7.0%) vs. 8/94 (8.5%); P = 1.000).

Baseline laboratory measurements are shown in Table 2. Serum total protein (6.2 ± 0.6 vs. 6.4 ± 0.6 g/dL; P = 0.049), albumin (2.6 ± 0.8 vs. 3.2 ± 0.8 g/dL; P < 0.001), and phosphorus (4.6 ± 1.2 vs. 5.9 ± 1.8 mg/dL; P < 0.001) were lower in the older group. Other laboratory variables did not differ significantly between groups.

**Table 2:** Blood test results of patients receiving hemodialysis in December 2017: Comparison of patients aged 75 years or older with younger patients. Data are presented as mean ± SD. P-values were calculated using Student’s t-test.

Variable	≥75 years old (n = 57)	<75 years old (n = 94)	Test statistic	P value
Hemoglobin, g/dL	10.7 ± 1.3	11.0 ± 1.6	t = -1.20	0.234
Serum total protein, g/dL	6.2 ± 0.6	6.4 ± 0.6	t = -1.99	0.049
Serum albumin, g/dL	2.6 ± 0.8	3.2 ± 0.8	t = -4.47	<0.001
Total cholesterol, mg/dL	142.4 ± 40.2	150.3 ± 36.9	t = -1.23	0.22
Corrected calcium, mg/dL	9.0 ± 0.7	9.0 ± 0.6	t = 0.00	1
Parathyroid hormone, pg/mL	126.9 ± 102.7	160.4 ± 132.5	t = -1.63	0.104
Serum iron, mg/dL	55.8 ± 25.2	64.5 ± 27.9	t = -1.93	0.056
Ferritin, ng/mL	153.6 ± 170.5	107.7 ± 125.5	t = 1.90	0.06
Serum phosphorus, mg/dL	4.6 ± 1.2	5.9 ± 1.8	t = -4.84	<0.001

Cause-specific characteristics among patients who died are summarized descriptively in Table 3. Patients who died from cerebrovascular/cardiovascular disease were younger on average than those who died from renal failure or infection. Of the 12 patients who died from cerebrovascular/cardiovascular causes, 8 (66.7%) were younger than 75 years, and four of the five acute myocardial infarction deaths occurred in patients younger than 75 years (Table 4).

**Table 3:** Deaths among patients receiving hemodialysis were classified by cause during the five-year follow-up period: renal failure, infectious diseases, CCD, other causes, and unknown causes. Data are descriptive because of the small subgroup sample sizes. CCD: Cerebrovascular/cardiovascular disease.

Variable	Renal failure (n = 18)	Infection (n = 16)	CCD (n = 12)	Other causes (n = 7)	Unknown causes (n = 2)
Age, years	77.2 ± 8.9	76.4 ± 12.3	65.0 ± 10.8	81.9 ± 7.2	80.5 ± 9.2
BMI, kg/m²	19.7 ± 3.3	20.5 ± 3.5	25.1 ± 8.6	22.5 ± 1.9	19.8 ± 1.1
Hemodialysis duration, months	110 ± 124	152 ± 145	71 ± 48	106 ± 131	39 ± 31
Diabetes mellitus	6/18 (33.3%)	3/16 (18.8%)	6/12 (50.0%)	2/7 (28.6%)	1/2 (50.0%)
Nephrogenic diseases	12/18 (66.7%)	13/16 (81.3%)	6/12 (50.0%)	5/7 (71.4%)	1/2 (50.0%)

**Table 4:** Characteristics of patients who died from cerebrovascular or cardiovascular diseases during hemodialysis. Cases 1-4 were aged ≥75 years; cases 5-12 were aged <75 years. HD: Hemodialysis.

Case	Age	Sex	Cause of renal failure	HD duration, months	Major comorbidities	Cause of death
1	76	Male	Diabetes mellitus	111	Rectal cancer	Acute myocardial infarction
2	76	Female	Nephrogenic disease	18	Hypertension	Acute subdural hematoma
3	78	Female	Nephrogenic disease	67	Aortic stenosis	Cardiogenic cerebral embolism
4	79	Male	Nephrogenic disease	27	Hypertension	Cerebral infarction
5	48	Male	Nephrogenic disease	6	Angina pectoris	Cerebral hemorrhage
6	50	Male	Diabetes mellitus	39	Angina pectoris	Acute lower-extremity arterial occlusion
7	59	Female	Nephrogenic disease	125	Thoracic aortic aneurysm	Aortic dissection
8	58	Male	Diabetes mellitus	31	Hypertension and hyperlipidemia	Acute myocardial infarction
9	59	Male	Diabetes mellitus	117	Angina pectoris	Acute myocardial infarction
10	63	Male	Diabetes mellitus	117	Aortic stenosis	Acute myocardial infarction
11	64	Female	Nephrogenic disease	50	Hypertension	Aortic dissection
12	70	Male	Diabetes mellitus	139	Atrial fibrillation and hepatitis C	Acute myocardial infarction

Eleven patients developed malignant disease during follow-up: 7/57 (12.3%) in the older group and 4/94 (4.3%) in the younger group. Among these patients, 6/7 (85.7%) in the older group died during the observation period, whereas 0/4 (0%) in the younger group died during follow-up (Table 5). These cancer-related findings were descriptive because of the small number of cases.

**Table 5:** Patients who developed malignant diseases during the five-year follow-up period while receiving hemodialysis. Cases 1-7 were aged ≥75 years; cases 8-11 were aged <75 years. HD: Hemodialysis.

Case	Age	Sex	Cause of renal failure	HD duration, months	Malignant disease	Illness period, months	Outcome
1	75	Male	Nephrogenic disease	46	Renal cancer	26	Died
2	75	Female	Nephrogenic disease	152	Bile duct cancer	17	Alive
3	81	Male	Nephrogenic disease	14	Rectal cancer	53	Died
4	82	Female	Nephrogenic disease	49	Parotid cancer	5	Died
5	89	Male	Diabetes mellitus	30	Lung cancer	56	Died
6	90	Male	Nephrogenic disease	50	Lung cancer	1	Died
7	91	Female	Nephrogenic disease	91	Lung cancer	2	Died
8	48	Male	Diabetes mellitus	48	Renal cancer	42	Alive
9	67	Female	Nephrogenic disease	356	Renal and breast cancers	53	Alive
10	70	Female	Nephrogenic disease	9	Rectal cancer	8	Alive
11	71	Female	Nephrogenic disease	83	Pancreatic cancer	9	Alive

## Discussion

This five-year single-center observational study had several clinically relevant findings. Patients aged 75 years or older had substantially higher cumulative all-cause mortality than younger patients. Deaths attributed to renal failure and infection were also more frequent in the older group, whereas cerebrovascular/cardiovascular deaths were not more frequent in the older group and occurred largely among younger patients. Older patients had more upper-body fractures and lower baseline serum albumin and phosphorus levels. These findings indicate that, in this regional hospital cohort, older hemodialysis patients had a vulnerability profile characterized by high mortality, fracture risk, and possible nutritional or inflammatory burden.

The higher mortality observed in the older group is consistent with previous registry and cohort studies showing worse prognosis with advancing age among patients receiving maintenance hemodialysis [1-3]. However, the present results should not be interpreted as evidence that chronological age alone caused the mortality difference. Frailty, functional impairment, comorbidity burden, inflammation, dialysis adequacy, treatment preferences, and nutritional status were not fully captured [5-13]. The findings are therefore best interpreted as real-world descriptive evidence that patients aged 75 years or older require careful risk assessment and individualized care planning.

The greater frequency of upper-body fractures in the older group is clinically important. In older dialysis patients, upper-extremity fractures may reflect falls, impaired balance, sarcopenia, frailty, and chronic kidney disease-mineral and bone disorder [7-9, 14-21]. Such fractures can also impair activities of daily living and may complicate vascular access management when the affected limb contains an arteriovenous fistula or graft. Regional dialysis centers with access to orthopedic care may therefore be well positioned to implement fall-risk screening, strength and balance interventions, medication review, and coordinated bone-health management [14-21].

Lower serum albumin and phosphorus levels in the older group should not be interpreted simply as favorable phosphate control. In elderly hemodialysis patients, low phosphorus may reflect reduced dietary intake, protein-energy wasting, inflammation, or frailty [10-13]. Similarly, hypoalbuminemia may indicate nutritional vulnerability or inflammatory burden [10-13]. These findings support individualized nutritional assessment rather than uniform intensification of phosphate restriction, especially when dietary restriction could reduce protein intake [10, 11].

GI bleeding did not differ significantly by age in this cohort, but it remains an important dialysis-related complication. Systematic evidence indicates that chronic kidney disease is associated with increased GI bleeding risk and adverse outcomes, and population-based data suggest that hemodialysis may be associated with a higher risk of GI bleeding-related hospitalization than peritoneal dialysis in some settings [22, 23]. Therefore, endoscopy-confirmed bleeding events in regional cohorts should be interpreted in the context of medication exposure, anticoagulation practices, gastrointestinal comorbidity, and competing mortality risks.

Cerebrovascular/cardiovascular deaths appeared to occur more often among relatively younger patients, including several acute myocardial infarction deaths. This pattern may reflect competing risks, survivorship effects, dialysis vintage, or differences in cardiovascular risk profiles; older patients may have died from infection, renal failure, or malignancy before experiencing cardiovascular death [2, 3]. Because formal time-to-event and competing-risk analyses were not performed, this finding should be regarded as hypothesis-generating.

Infection-related mortality was also more frequent among older patients in the present cohort. This is clinically plausible because infection remains a major noncardiovascular cause of death in dialysis populations; recent registry studies show that infection-related mortality persists despite improvements over time and is higher in older dialysis patients [24, 25]. These findings support attention to vaccination, vascular access management, early recognition of pneumonia or sepsis, and individualized decisions about hospitalization and supportive care.

The malignancy findings were also descriptive but clinically noteworthy. More patients in the older group developed cancer during follow-up, and most older patients with cancer died during the observation period. Dialysis populations have been reported to have increased cancer incidence and cancer-related mortality, including higher risks of several urological cancers, although risk estimates vary by cancer site, age, dialysis duration, transplant eligibility, and competing mortality [26-29]. The higher mortality among older patients with cancer may reflect frailty, limited physiologic reserve, differences in cancer stage, treatment tolerance, cancer-screening intensity, or access to standard oncologic regimens. The small number of cases in the present study precludes firm conclusions, but the finding supports the need for coordination among nephrology, oncology, geriatrics, and palliative/supportive care when malignancy is diagnosed in older dialysis patients.

The strengths of this study include complete enumeration of maintenance hemodialysis patients at a regional core hospital, five-year longitudinal follow-up, and clinically confirmed ascertainment of GI bleeding and fractures. The single-center design also provided relatively consistent dialysis practice, laboratory monitoring, and clinical documentation. These strengths are balanced by important limitations. This was a retrospective, single-center prevalent-cohort study, so generalizability is limited and survivor bias is possible. Age group was defined only at baseline, and patients managed conservatively without dialysis were not included. Event ascertainment depended on routine documentation, fractures were counted only when clinically recognized and imaging-confirmed, and cause of death was not adjudicated by an independent blinded committee. Mortality was analyzed as cumulative mortality rather than time-to-event data, and competing risks could not be quantified. The sample size and number of cause-specific deaths and malignancies were small, and adjustment for frailty, functional status, standardized comorbidities, inflammation, dialysis adequacy, medication exposure, nutritional intake, socioeconomic factors, and treatment preferences was not possible. Baseline laboratory values were based on a single time point, and no robust imputation or multiple-comparison adjustment was performed. Thus, all findings should be interpreted as descriptive and hypothesis-generating.

## Conclusions

In this retrospective cohort of 151 maintenance hemodialysis patients treated at a single regional Japanese hospital, five-year all-cause mortality was significantly higher in the older group than in the younger group (59.6% vs. 22.3%, P < 0.001). The older group also had more frequent upper-body fractures and lower serum albumin and phosphorus levels than the younger group. Deaths attributed to renal failure and infection were more frequent in the older group, whereas cerebrovascular/cardiovascular deaths were observed mainly among younger patients. These findings meet the study objective of describing age-stratified outcomes in this regional hemodialysis population but should be interpreted as associations rather than causal effects.

Clinically, the results suggest that older hemodialysis patients may benefit from individualized assessment of nutrition, frailty, fall and fracture risk, infection vulnerability, and cancer-related supportive needs. Prospective multicenter studies incorporating standardized geriatric and nutritional assessment, medication data, survival analysis, and competing-risk methods are needed to validate these findings and define targeted interventions for this vulnerable population.
